# Dataset on the evaluation of chemical and mechanical properties of steel rods from local steel plants and collapsed building sites

**DOI:** 10.1016/j.dib.2018.10.162

**Published:** 2018-11-02

**Authors:** A.A. Adeleke, J.K. Odusote, P.P. Ikubanni, O.A. Lasode, O.O. Agboola, A. Ammasi, K.R. Ajao

**Affiliations:** a Department of Mechanical Engineering, University of Ilorin, Kwara State, Nigeria; b Department of Materials and Metallurgical Engineering, University of Ilorin, Kwara State, Nigeria; c Department of Mechanical Engineering, Landmark University, Omu-Aran, Kwara State, Nigeria; d Metal Extraction and Recycling Division, National Metallurgical Laboratory, Jamshedpur, India

**Keywords:** Mechanical properties, Collapsed building, Steel rods, Chemical compositions, Percentage elongation

## Abstract

The quality of steel rods used in structural applications has been subjected to continuous scrutiny by researchers in Nigeria. In this data article, the experimental data on the chemical and mechanical properties of steel rods from collapsed building sites and local steel plants have been reported. The chemical composition consisting of carbon, manganese, silicon, sulphur, phosphorus among other elements were recorded using an optical emission spectrometer. Some of the main elements were used to evaluate the carbon equivalent value and the results are reported in this article. The yield strength, ultimate tensile strength and percentage elongation were also presented as obtained from the universal testing machine. The hardness values of the steel rod samples were also presented.

**Specification table**

**Table:** 

Subject area	Mechanical Engineering
More specific subject area	Materials and metallurgy, Iron and steel, Construction materials
Type of data	Tables, figures and pictures
How data was acquired	Steel rods from local steel plants and collapsed building sites were subjected to chemical composition analyses using optical emission spectrometer, universal testing machine for yield strength, ultimate tensile strength, and percentage elongation with hardness tester.
Data format	Raw, processed and analysed.
Experimental factors	Triplicate samples were used for the various tests and the average has been reported.
Experimental features	Steel rods from different collapsed sites and local plants with various diameters.
Data source location	Strength of Materials laboratory, Landmark University; Materials and metallurgical laboratory, Obafemi Awolowo University, Ile Ife and University of Ilorin, Nigeria.
Data accessibility	Data are as presented in this article
Related research articles	[1] Odusote J.K. and Adeleke A.A. (2012), Analysis of properties of reinforcing steel bars: Case study of collapsed buildings in Lagos, Nigeria, Applied Mechanics and Materials, 204–208:3052-3056.
	[2] Adeleke A.A. and Odusote J.K. (2013), Evaluation of mechanical properties of reinforcing steel bars from collapsed buildings sites, Journal of Failure Analysis and Prevention, 13:737-743.
	[3] Ikubanni P.P., Adediran A.A., Adeleke A.A. Ajao K.R. and Agboola O.O. (2017), Mechanical properties improvement evaluation of medium carbon steel in different media, International Journal of Engineering Research in Africa, 32:1-10.

**Value of the data**

• The data provide information on the utilization of scraps as a raw material in the production of reinforcing steel rods.
• The reported data reveal the chemical and mechanical properties of steel rods from local plants and collapsed sites and this will serve as bases for further study on reinforcing steel bars produced from scraps and their contributions to collapse of building structures.
• The data presented are useful to construction Engineers within the country when selecting steel rods for structural reinforcement.

## 1 Data

The data presented information on the chemical compositions, carbon equivalent value (CEV), yield strength (YS), ultimate tensile strength (UTS), hardness value and percentage elongation of steel rods (different diameters) from collapsed building sites, and local plants. The chemical (elemental) compositions of the steel rods are presented in Table 1 containing carbon (C), silicon (Si), manganese (Mn), sulphur (S), and phosphorus (P) among others. Table 2 shows the yield strength (YS), while Table 3 represents the ultimate tensile strength (UTS) of the steel rod samples. The percentage elongation of the steel rod samples are also presented in Table 4. Table 5 shows the carbon equivalent and the hardness values for the steel rod samples.

**Table 1:** Elemental (Chemical) composition of the steel rod samples.

**Element (%)**	**Samples**										
	**EA**	**IA**	**SA**	**OA**	**AA**	**ALA**	**A12**	**A16**	**B10**	**B12**	**B16**
**C**	0.339	0.311	0.345	0.324	0.351	0.315	0.259	0.329	0.330	0.169	0.291
**Si**	0.231	0.223	0.206	0.222	0.23	0.216	0.179	0.176	0.307	0.228	0.193
**S**	0.080	0.086	0.079	0.018	0.049	0.063	0.038	0.036	0.040	0.047	0.042
**P**	0.069	0.079	0.068	0.075	0.067	0.056	0.043	0.042	0.045	0.056	0.054
**Mn**	0.983	0.991	0.806	1.001	0.963	0.981	0.519	0.555	0.727	0.579	0.579
**Ni**	0.106	0.107	0.110	0.112	0.121	0.123	0.100	0.112	0.091	0.085	0.105
**Cr**	0.223	0.223	0.225	0.212	0.220	0.205	0.154	0.164	0.163	0.204	0.271
**Mo**	0.030	0.030	0.031	0.023	0.031	0.032	0.028	0.023	0.027	0.030	0.025
**V**	0.006	0.006	0.006	0.005	0.005	0.048	0.005	0.004	0.004	0.005	0.004
**Cu**	0.283	0.284	0.282	0.281	0.283	0.281	0.342	0.261	0.245	0.292	0.308
**W**	0.012	0.012	0.011	0.013	0.011	0.012	0.010	0.013	0.015	0.012	0.011
**Ti**	0.002	0.002	0.002	0.002	0.002	0.002	0.001	0.002	0.003	0.001	0.003
**Sn**	0.016	0.016	0.015	0.013	0.016	0.017	0.014	0.015	0.017	0.016	0.013
**Co**	0.011	0.011	0.011	0.012	0.011	0.011	0.012	0.010	0.014	0.013	0.011
**Al**	0.006	0.003	0.004	0.005	0.005	0.003	0.021	0.021	0.021	0.020	0.021
**Nb**	0.001	0.001	0.001	0.001	0.001	0.001	0.002	0.001	0.001	0.002	0.001
**Fe**	97.602	97.615	97.618	97.681	97.634	97.634	98.273	98.236	97.950	98.241	98.068

**Table 2:** Yield strength of the steel rod samples.

**YS (N/mm**^**2**^**)**				
	**Sample 1**	**Sample 2**	**Sample 3**	**Average**
**EA**	460.16	458.12	462.15	460.14
**IA**	488.11	488.56	482.28	486.32
**SA**	550.66	552.01	552.02	551.56
**OA**	493.21	491.06	492.04	492.10
**AA**	468.04	467.20	466.58	467.27
**ALA**	466.94	465.98	467.91	466.94
**A12**	406.10	406.63	404.34	405.69
**A16**	389.04	388.19	387.12	389.12
**B10**	410.89	409.92	410.96	410.59
**B12**	406.44	404.98	402.44	404.62
**B16**	373.04	372.15	374.21	373.13

**Table 3:** Ultimate tensile strength of the steel rod samples.

**UTS (N/mm**^**2**^**)**				
	**Sample 1**	**Sample 2**	**Sample 3**	**Average**
**EA**	598.88	596.28	596.48	597.21
**IA**	586.53	585.86	584.92	585.77
**SA**	625.39	628.13	623.74	625.75
**OA**	556.55	556.76	556.98	556.76
**AA**	545.69	545.74	545.44	545.62
**ALA**	577.48	578.55	576.22	576.42
**A12**	584.00	581.28	582.03	582.44
**A16**	590.06	592.00	591.01	591.02
**B10**	668.14	668.02	667.02	667.73
**B12**	544.88	545.74	543.80	544.80
**B16**	556.58	555.89	555.98	556.14

**Table 4:** The percentage elongation of the steel rod samples.

%Elongation				
	**Sample 1**	**Sample 2**	**Sample 3**	**Average**
**EA**	9.08	9.18	8.80	9.02
**IA**	11.92	12.26	10.98	11.72
**SA**	9.94	9.93	10.08	9.80
**OA**	10.00	10.01	10.30	10.1
**AA**	10.16	10.18	10.01	10.11
**ALA**	9.87	9.48	10.34	9.89
**A12**	30.96	31.67	31.64	31.42
**A16**	29.22	28.11	26.52	27.95
**B10**	26.08	28.07	27.17	27.11
**B12**	32.11	30.65	31.88	31.54
**B16**	29.68	32.08	29.51	30.42

**Table 5:** The hardness and carbon equivalent values (CEV) of the steel rod samples.

**Samples**	**CEV**	**Hardness (HRC)**
**EA**	0.720	21.19 ± 1.10
**IA**	0.692	20.22 ± 0.98
**SA**	0.677	19.63 ± 1.00
**OA**	0.704	20.45 ± 1.12
**AA**	0.727	21.23 ± 0.80
**ALA**	0.698	20.34 ± 0.85
**A12**	0.500	18.04 ± 1.20
**A16**	0.575	18.21 ± 1.11
**B10**	0.650	20.71 ± 1.22
**B12**	0.444	16.83 ± 0.88
**B16**	0.572	17.05 ± 1.44

## 2 Experimental design, materials, and methods

**Fig. 1:**
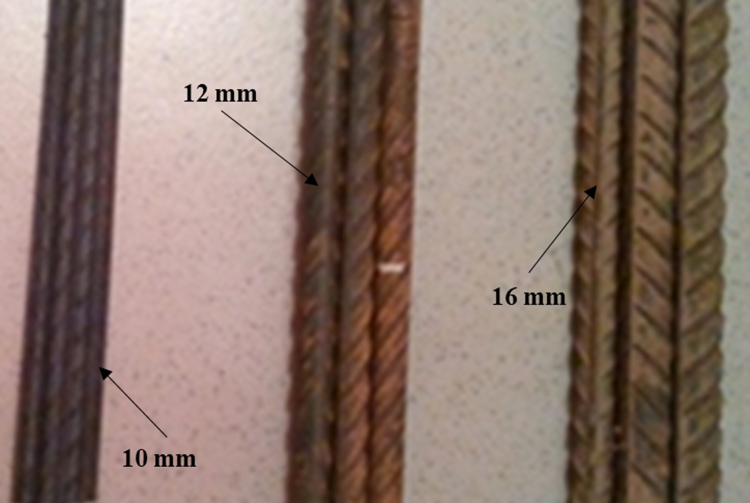
Steel rod samples of different diameters.

Steel rod samples of 12 mm diameter were collected from six different collapsed building sites in Lagos, Nigeria. These sites include a 2-storey building at Ewutu area (EA), 3-storey building under construction at Ilesanmi area (IA), 3-storey building at Ojo area (OA), a storey building at Agege area (AA), two- storey building Alagbado area (ALA) and a storey building at Sango area (SA). Five other samples of steel rod were obtained from two steel plants in Osun State, Nigeria. Steel rods of 12 and 16 mm diameters were collected from steel plant A and tagged as A12 and A16, respectively. Steel rods of 10, 12, and 16 mm were collected from steel plant B and tagged as B10, B12, and B16, respectively. Examples of these rods are shown in Fig. 1.

**Fig. 2:**
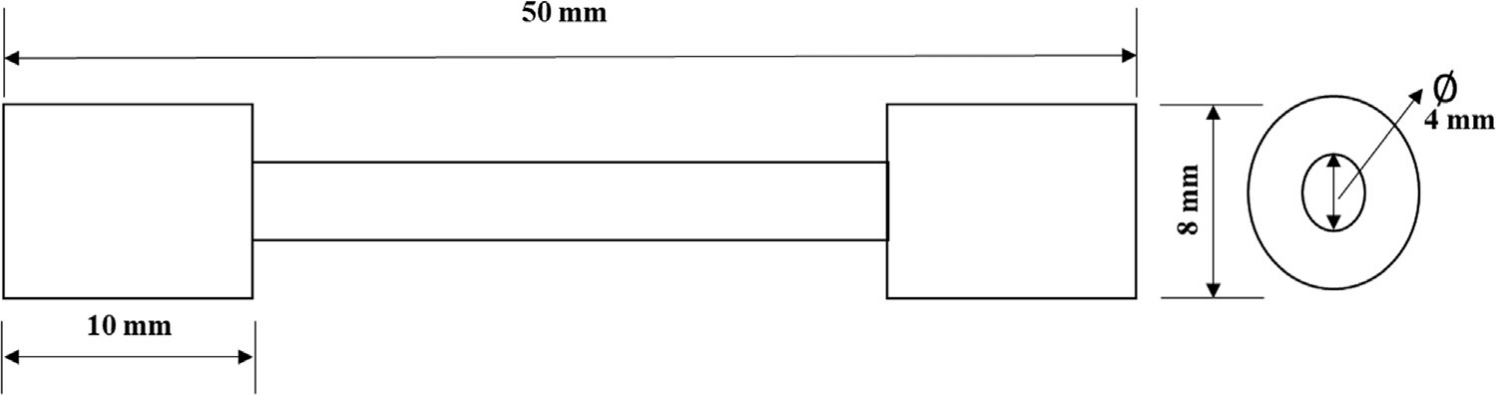
Tensile test specimen from the steel rods.

**Fig. 3:**
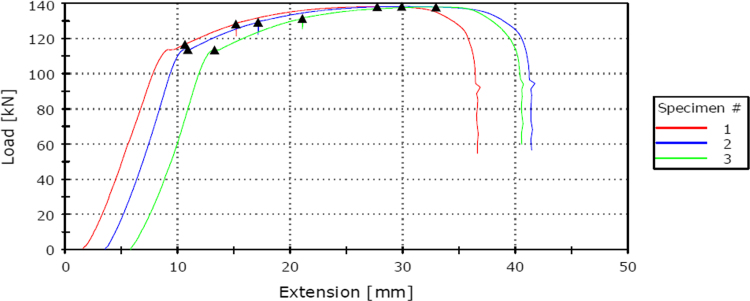
Load to extension graph of the steel rod under tensile test (three replicates).

Optical emission spectrometer (Model RLMA, Serial No 871) was used for chemical composition analyses. Steel rod sample was sectioned into small sizes after which it was then ground using an industrial grinding machine with a 60-grit emery paper to relatively smooth surface. The polished sample was charged with argon gas so as to remove dirt or impurities. Argon gas was used because it is 99.9% inert gas (very pure and non-reactive). The sample was then placed in the optical emission with a monitor in order to determine the elemental compositions in mass percentage. Carbon equivalent value was estimated from some major elements using the relationship in Eq. (1). The tensile test for the samples was carried out using Instron Universal Tensile Testing Machine (Model Number 5569). The test specimens were cut to sizes as shown in Fig. 2 to grip properly to the jaw of the machine. During the test, the machine jaws were pulled apart automatically until the specimen necks eventually broken or fractured. As the load was slowly applied on the tensile specimen, load to extension graphs were continually plotted as shown in Fig. 3. The ultimate tensile strength (UTS) was evaluated from Fig. 3 using Eq. (2). Similarly, the yield strength (YS) was calculated using Eq. (3). The two ends of the fractured samples were fitted together to obtain the final gage length as well as the diameter using venier calliper. The percentage elongation was determined using Eq. (4). Hardness of the samples was determined on a Brinell hardness tester (Model HBW, Serial no: VR-44, FOUNDRAX) with indenter with a diameter of 10 mm and load of 500 kg. The Brinnel hardness number was converted and reported as Rock well value (HRC).

(1) CEV=%C+(%Mn+%Si)/6+(%Cr+%Mo+%V)/5+(%Cu+%Ni)/15

(2) $$\mathrm{UTS}\;=\;M_{L}/\mathrm{CSA}$$

(3) $$\mathrm{YS}\;=\;Y_{L}/\mathrm{CSA}$$

(4) $$\%\mathrm{Elongation}\;=\;{(L}_{f}-L_{o})/L_{o}$$

where %C is the percentage carbon content, %Mn is percentage manganese content, %Si is percentage silicon content, %Cr is percentage chromium content, %Mo is percentage molybdenum content, %V is percentage vanadium content, %Cu is percentage copper content, %Ni is percentage nickel content, $M_{L}$ is the maximum load (kN), $Y_{L}$ is yield load (kN), CSA is cross-sectional area (mm^2^), $L_{f}$ is the final gauge length (mm) and $L_{o}$ is the initial length (mm).
